# Comparative transcriptome analysis of latex from rubber tree clone CATAS8-79 and PR107 reveals new cues for the regulation of latex regeneration and duration of latex flow

**DOI:** 10.1186/s12870-015-0488-3

**Published:** 2015-04-18

**Authors:** Jinquan Chao, Yueyi Chen, Shaohua Wu, Wei-Min Tian

**Affiliations:** Ministry of Agriculture Key Laboratory of Biology and Genetic Resources of Rubber Tree/ State Key Laboratory Breeding Base of Cultivation and Physiology for Tropical Crops, Rubber Research Institute, Chinese Academy of Tropical Agricultural Sciences, Danzhou, Hainan 571737 PR China

**Keywords:** *Hevea brasiliensis* Muell. Arg, RNA-Seq, Transcriptome, Latex regeneration, Duration of latex flow

## Abstract

**Background:**

Rubber tree (*Hevea brasiliensis* Muell. Arg.) is the primarily commercial source of natural rubber in the world. Latex regeneration and duration of latex flow after tapping are the two factors that determine rubber yield of rubber tree, and exhibit a huge variation between rubber tree clones CATAS8-79 and PR107.

**Results:**

To dissect the molecular mechanism for the regulation of latex regeneration and duration of latex flow, we sequenced and comparatively analyzed latex of rubber tree clone CATAS8-79 and PR107 at transriptome level. More than 26 million clean reads were generated in each pool and 51,829 all-unigenes were totally assembled. A total of 6,726 unigenes with differential expression patterns were detected between CATAS8-79 and PR107. Functional analysis showed that genes related to mass of categories were differentially enriched between the two clones. Expression pattern of genes which were involved in latex regeneration and duration of latex flow upon successive tapping was analyzed by quantitative PCR. Several genes related to rubber biosynthesis, cellulose and lignin biosynthesis and rubber particle aggregation were differentially expressed between CATAS8-79 and PR107.

**Conclusions:**

This is the first report about probing latex regeneration and duration of latex flow by comparative transcriptome analysis. Among all the suggested factors, it is more important that the level of endogenous jasmonates, carbohydrate metabolism, hydroxymethylglutaryl-CoA reductase (HMGR) and *Hevea* rubber transferase (HRT) in mevalonate (MVA) parthway for latex regeneration while the level of endogenous ethylene (ETH), lignin content of laticifer cell wall, antioxidants and glucanases for the duration of latex flow. These data will provide new cues for understanding the molecular mechanism for the regulation of latex regeneration and duration of latex flow in rubber tree.

**Electronic supplementary material:**

The online version of this article (doi:10.1186/s12870-015-0488-3) contains supplementary material, which is available to authorized users.

## Background

Rubber tree (*Hevea brasiliensis* Muell. Arg.) is the main source of natural rubber [1-4]. The natural rubber is synthesized and stored in laticifer, a specific tissue densely located in the secondary phloem of trunk [5]. By successive tapping, white or yellowish milky latex is expelled and collected. The latex is the cytoplasm of laticifer cells and used to refine natural rubber. It contains numerous rubber particles and lutoids as well as general eukaryotic organelles [6].

Latex regeneration and duration of latex flow after tapping are important factors that determine rubber yield of rubber tree. Sucrose, water and nitrogen sources supplying from the surrounding parenchyma cells act as raw materials for latex regeneration between two tappings [7,8]. Isopentenyl pyrophosphate (IPP) is the direct precursor for rubber biosynthesis and mainly derived from the MVA pathway although 2-C-methyl-D-erythritol 4-phosphate (MEP) pathway is suggested to be an alternative source [6]. Catalyzing by enzymes as prenyltransferase, the rubber transferase, IPP initiates the subsequent extensive prenyl chain elongation process for the formation of rubber macromolecules. It is well known that tapping can promote latex regeneration and there is obvious difference in the rubber content of latex among varieties upon ethrel stimulation [7]. The duration of latex flow is influenced by various factors, such as laticifer turgor, plug formation at the end of severed laticifer, and ethrel application. Plugging of severed laticifer end leads to the termination of latex flow from the wounded site of rubber trees and has been a key limiting factor for the yield of *Hevea* [9,10]. It is widely believed that the severed laticifers are plugged by rubber coagulum as a result of rubber particle aggregation (RPA) caused by the bursting of lutoids [11,12]. Inclusions and debris of lutoids from the burst lutoids are effective in rubber particle aggregation [13,14].

With the sequence technology development, digital gene expression tag profiling recently displays huge potential for exploring biological process [15-22]. By using next-generation massively parallel sequencing technologies, Triwitayakorn *et al*. sequenced 2,311,497 reads from rubber tree vegetative shoot apex transcriptome, generating 23 linkage groups covering 842.9 cM with a mean interval of 11.9 cM per linkage group [23]. Xia *et al. de novo* assembled 48,768 unigenes from transcriptome data of leave and latex of rubber tree in an effort to facilitate biological, biochemical and molecular researches on rubber biosynthesis [24]. Li *et al*. generated 22,756 unigenes from rubber tree bark transcritome and obtained 39,257 simple sequence repeats (SSRs) markers which may be benefit for marker-assisted selection in the cross breeding program of rubber tree [25]. Mantello *et al*. assembled 50,384 contigs with an average length of 400 bp from *H. brasiliensis* bark transcritome, and detected 17,927 SSRs and 404,114 single nucleotide polymorphisms (SNPs) [26]. In the present study, a comparative analysis of latex transcriptome between rubber tree clone PR107 and CATAS8-79 was performed to uncover the molecular mechanism for the regulation of latex regeneration and duration of latex flow.

## Results

### Difference in latex regeneration and duration of latex flow between rubber tree clone PR107 and CATAS8-79

**Figure 1:**
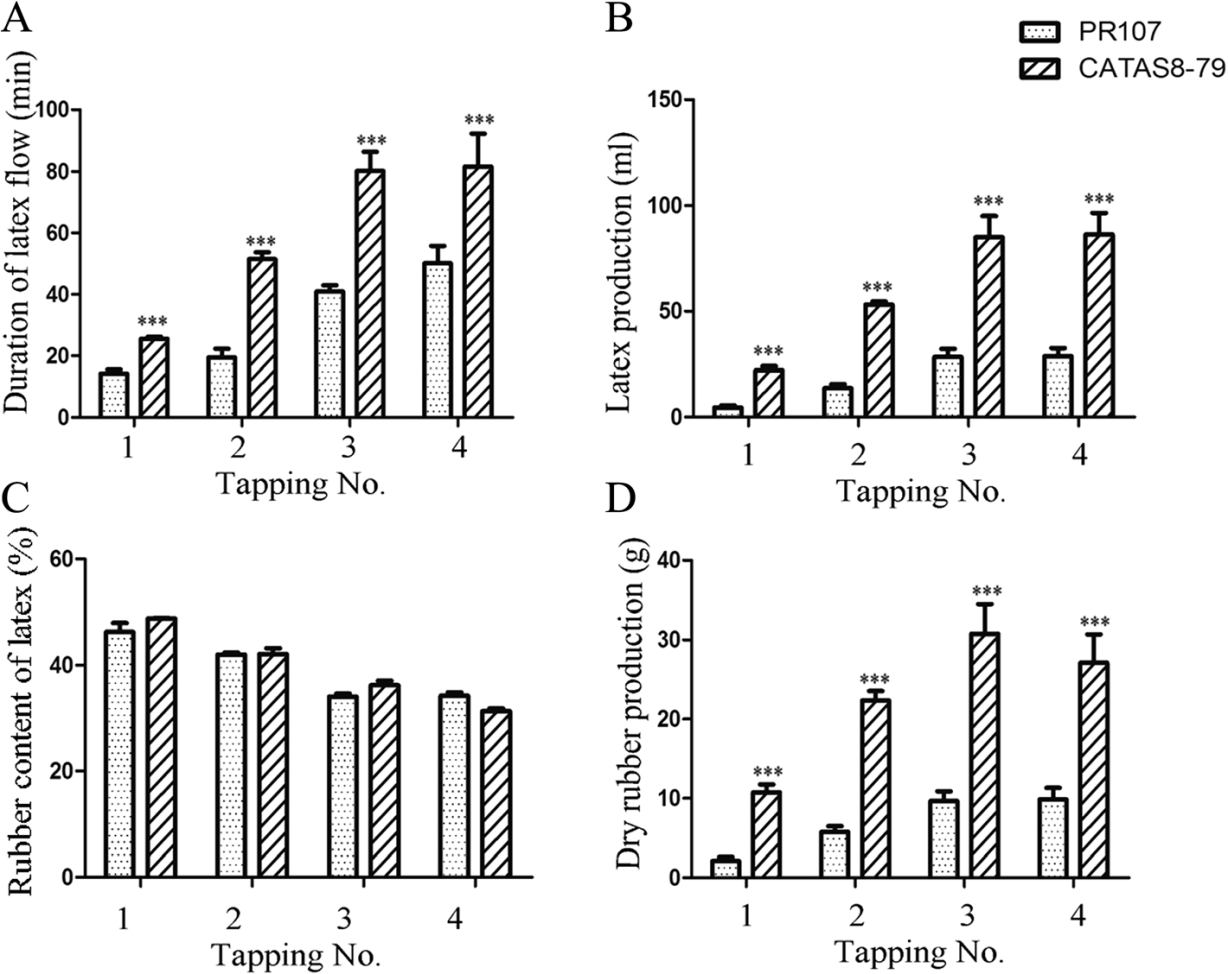
The difference in duration of latex flow **(A)**, latex regeneration **(B)**, rubber content of latex **(C)** and dry rubber production **(D)** between CATAS8-79 and PR107 upon successive tappings. Significant difference was indicated by the asterisks above the bars (***p < 0.01). Tapping No. represented Tapping Number.

Rubber tree clone CATAS8-79 and PR107 exhibited a huge difference in duration of latex flow (Figure 1A) and latex regeneration (Figure 1B) at each tapping. A total of about 260 ml of latex was gained from CATAS8-79 while only about 95 ml of latex from PR107 by four tappings (Figure 1B). The rubber content of latex, however, showed no difference between two clones at each tapping (Figure 1C). Consequently, rubber yield of CATAS8-79 was significantly higher than that of PR107 (Figure 1D), suggesting latex regeneration in CATAS8-79 was more effective than that in PR107 during the interval of successive tappings.

### Assembling, annotation of latex transcriptome

RNA were extracted from CATAS8-79 and PR107 at first tapping and sequenced with Illumina paired-end sequencing technology individually. After excluding low-quality reads such as empty adapters, 26 million clean reads were generated in each pool. Using SOAPdenove software, 296,736 and 308,262 contigs ranging from 100 bp to more than 3,000 bp were respectively assembled from CATAS8-79 and PR107 (NCBI accession numbers: GSE59981). By paired-end and gap-filling, contigs were further extended and finally assembled as a long sequence named “unigenes”. In this way, 53,571 and 57,806 unigenes were generated from CATAS8-79 and PR107 individually. The unigenes were further integrated into 51,829 all-unigenes with an average length of 640 bp and a N50 of 526 bp by paired-end joining (Table 1, Additional file 1: Table S1). The analyses following were carried out with all-unigenes (reffered to unigenes).

**Table 1:** ***de novo*** **assembly of** ***H. brasiliensis*** **transcriptome**

**Sample**	**Number**	**N50 (bp)**	**Mean (bp)**
CATAS8-79 unigene	53571	509	421
PR107 unigene	57806	427	375
All unigene	51829	640	526

**Figure 2:**
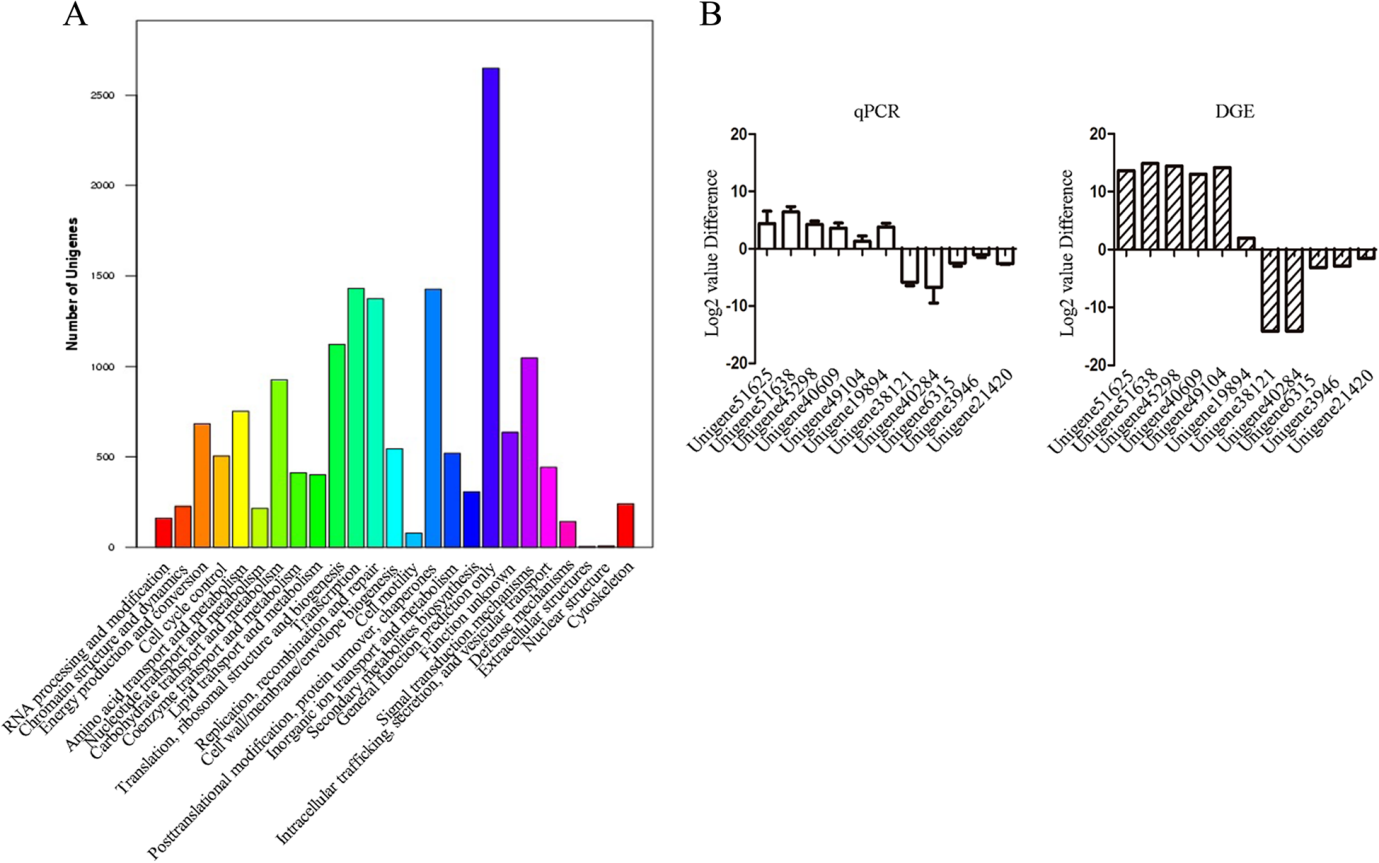
Classification and detection of differentially expressed unigenes. **(A)** Histogram of clusters of COG classifications of *H. brasiliensis* unigenes. Numbers on the Y-axis represent the uingenes numbers. **(B)** Validation of DGE by qRT-PCR. The left showed qRT-PCR and the right showed DGE data. Error bars for qRT-PCR show the standard deviation of three replicates. Unigene51625, BRASSINOSTEROID INSENSITIVE 1; Unigene51638, Cellulose synthase; Unigene45298, 4-coumarate-coa ligase; Unigene40609, aux/IAA protein; Unigene49104, beta-1,3-glucanase; Unigene19894, glutamine synthetase; Unigene38121, hydroxymethylglutaryl-CoA reductase; Unigene40284, Jasmonate O-methyltransferase; Unigene6315, 1-deoxy-D-xylulose 5-phosphate reductoisomerase; Unigene3946, 1-deoxy-D-xylulose 5-phosphate synthase; Unigene21420, Beta-amylase 2.

All the integrated unigenes were used to match against both the NCBI Non-redundant (Nr) and Swissprot protein databases using BLASTx program with an E-value threshold of 1E-5. Of which, 40,373 (77.9%) and 23,387 (45.12%) unigenes were positively matched with Nr protein database and Swissprot protein database, respectively (Additional file 2: Figure S1). Clusters of Orthologous Group (COG) analysis showed that 16,242 unigenes could be divided into 25 categories (Figure 2A). Of which, “General function predicted only” represented the largest group (2,647), followed by “Transcription” (1,431), and “Posttranslational modification, protein turnover, chaperones” (1,427). Categories of “Extracellular structures” and “Nuclear structure” only included 4 and 8 unigenes whereas 926 unigenes were classified into the category of “Carbohydrate transport and metabolism”.

By comparative analysis of transcriptome data of CATAS8-79 versus PR107 under condition of FDR ≤ 0.001 and |log2 Ratio| ≥ 1, there were 6,726 unigenes with differential expression. Of which, 3,018 were up-regulated while 3,708 were down-regulated. “Up-regulated” means the level of gene transcripts was higher in PR107 whereas “down-regulated” means the level of gene transcripts was higher in CATAS8-79. To validate the digital gene expression (DGE), 11 unigenes were selected to amplify by qRT-PCR in the latex samples from CATAS8-79 and PR107. These unigenes were different in abundance and expression pattern on the basis of DGE data. The expression pattern of most of them by qRT-PCR was similar to the corresponding DGE data (Figure 2B).

### Functional analysis of the enriched categories in CATAS8-79 and PR107

Kyoto Encyclopedia of Genes and Genomes (KEGG) analysis of all the differently expressed unigenes showed that mass of categories was differentially enriched between CATAS8-79 and PR107. Some of these differences may be related to the difference in latex regeneration and duration of latex flow between the two clones. The others may associate with the general difference in genetic background, considering that CATAS8-79 only contained one fourth descent of PR107.

**Figure 3:**
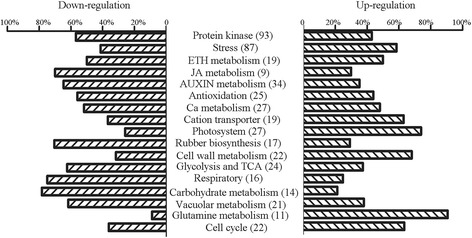
Histogram of metabolism categories enrichment in up-/down-regulation parts. Number in brackets showed corresponding unigenes detected in transcriptome.

**Figure 4:**
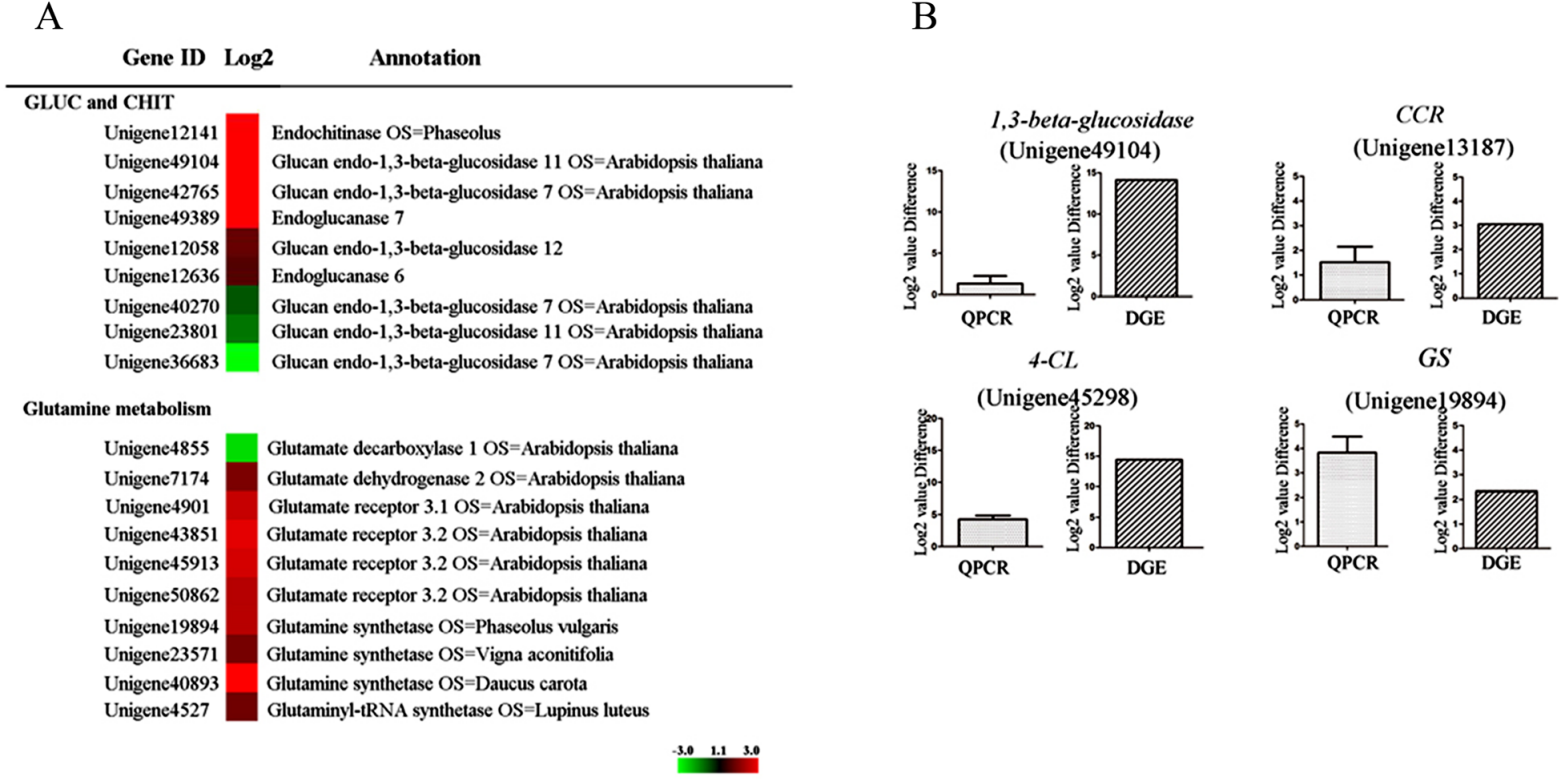
Differential expressed unigenes involving in cell wall biosynthesis, nitrogen metabolism and stress-related proteins. The left showed qRT-PCR **(A)** and the right showed heat map **(B)**. Error bars for qRT-PCR showed the standard deviation of three replicates. DGE values displayed as heat map. Colours bar represented expression levels of each gene which were either up-regulated (red) or down-regulated (blue).

Most of unigenes related to categories of stress-related proteins, cell wall biosynthesis and nitrogen requirement were significant up-regulated in PR107 (Figure 3). One unigene encoding for chitinase, eight unigenes for glucanase were detected in DGE data (Figure 4A), and unigene 49104 encoding beta-1,3-glucanase was analyzed by qRT-PCR (Figure 4B). Glutamate synthase (GS) plays a key role in integrating NH_4_ into amino acid, which is crucial for nitrogen requirement [27]. Recently, there is evidence that GS can directly regulate lignin biosynthesis and deposition in rice seeding [28]. Nine unigenes encoding for enzymes involving in glutamine metabolism were up-regulated in PR107 (Figure 4A), and one of them, unigene19894, was analyzed by qRT-PCR (Figure 4B). Several unigenes involving cellulose and lignin metabolism were widely activated in rubber clone PR107. Lignin is a component of cell wall and synthesized through shikimate pathway [29]. Unigenes encoding 4-coumarate-coa ligase (unigene45298, 4CL), cinnamoyl-CoA reductase (unigene13817, CCR), the key enzymes in shikimate pathway were analyzed by qRT-PCR (Figure 4B).

**Figure 5:**
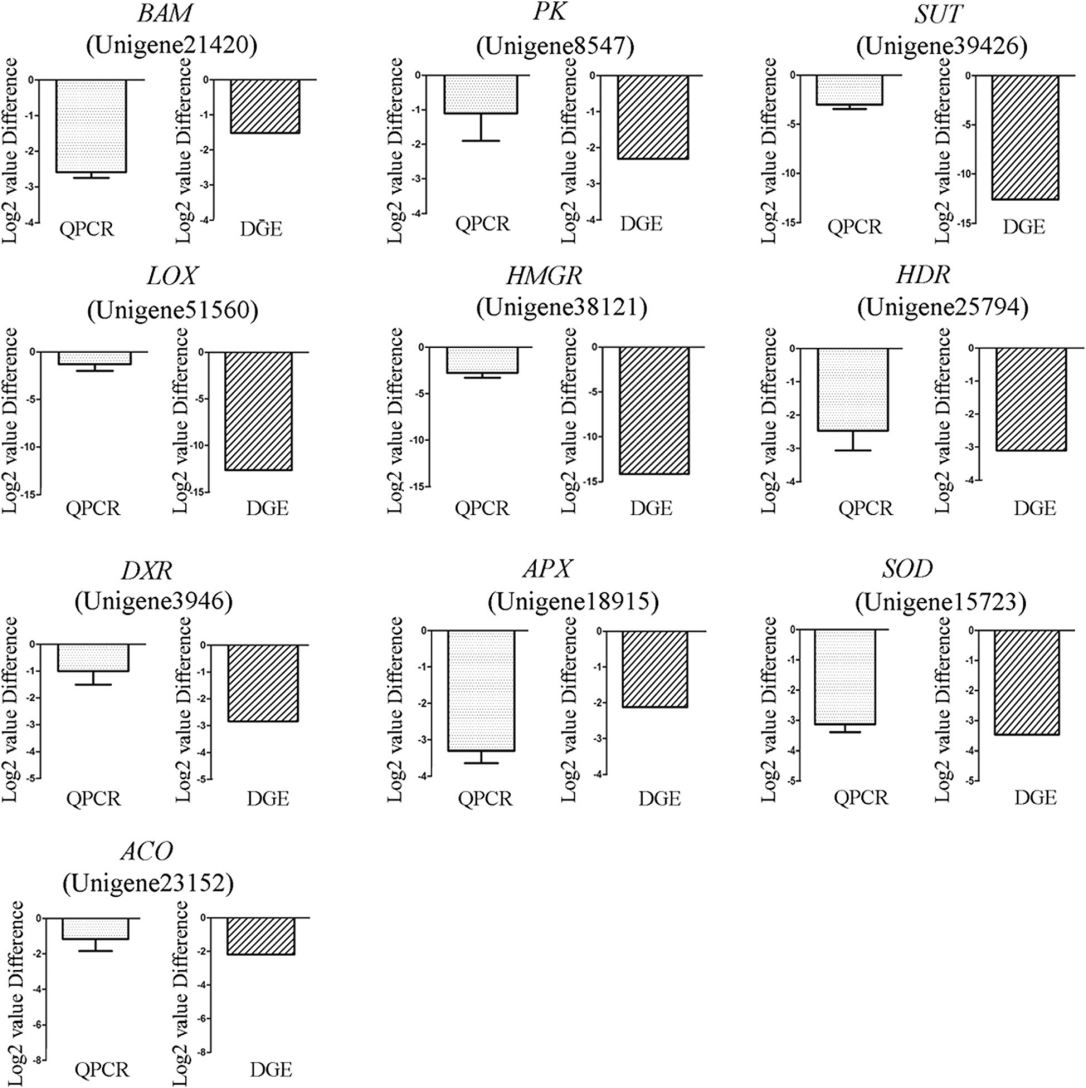
Differentially expressed unigenes involving hormone, carbohydrate and rubber biosynthesis processes. The left showed qRT-PCR and the right showed DGE data in each schematic. Error bars for qRT-PCR showed the standard deviation of three replicates.

**Figure 6:**
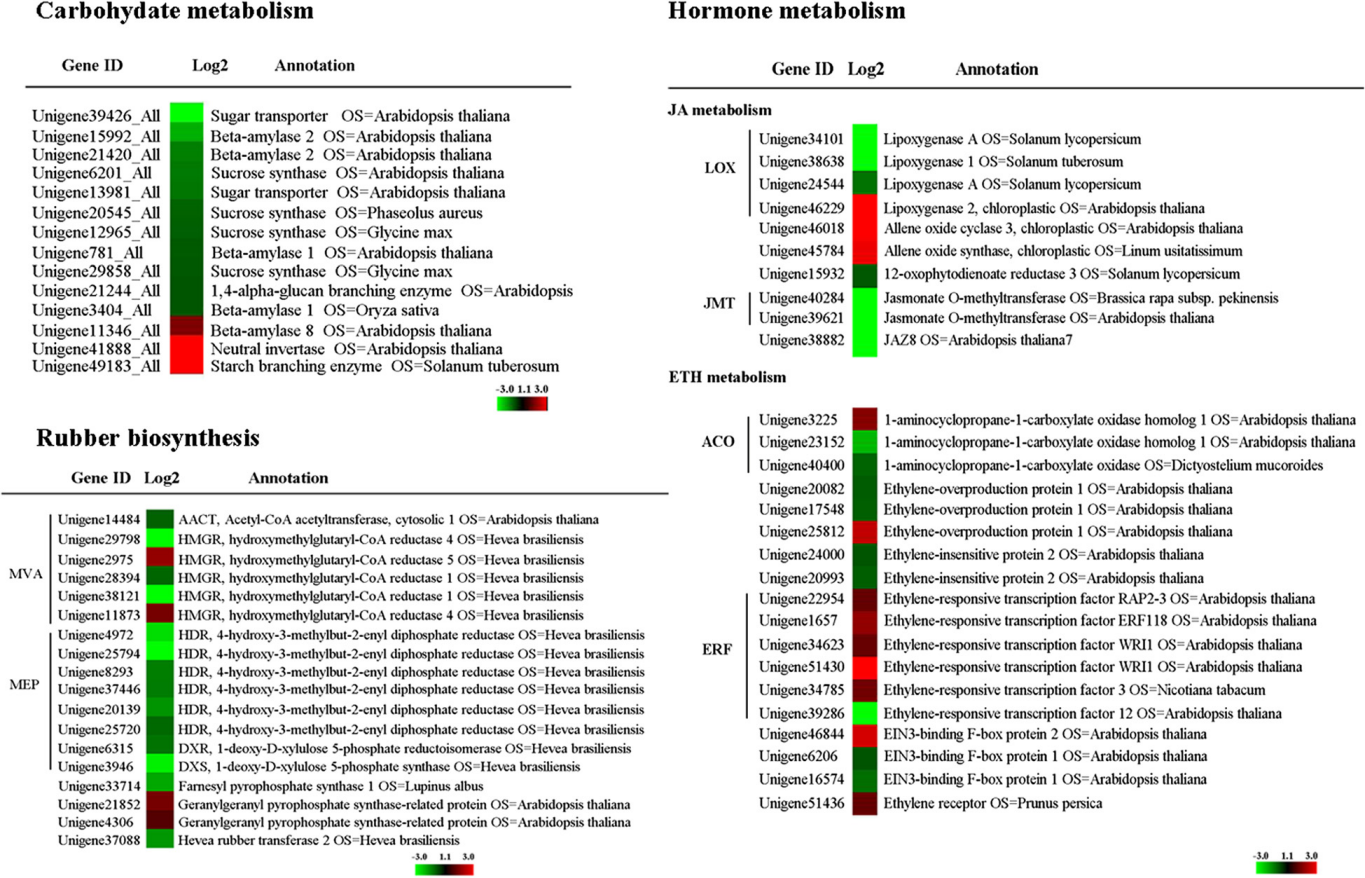
Heat map of genes involve in carbohydrate metabolism, rubber biosynthesis and hormone metabolism. DGE values displayed as heat map. Colours bar represented expression levels of each gene which were either up-regulated (red) or down-regulated (blue).

By contrast, most of unigenes related to categories of carbohydrate metabolism, rubber biosynthesis, hormone, and antioxidation were up-regulated in CATAS8-79 (Figure 3). Carbohydrate metabolism could provide carbon skeleton for the formation of various organic compounds [30,31]. DGE analysis showed that plenty of unigenes associating with carbohydrate metabolism were up-regulated, and the expression pattern of several unigenes mediating sucrose transportation (unigene39426 encoding sucrose transporter, SUT), starch degradation (unigene21420 encoding beta-amylase, BAM) and glycolysis (Unigene8547 encoding pyruvate kinase, PK) were analyzed by qRT-PCR (Figure 5). Both MVA and MEP pathways provide IPP, a precursor for the formation of final rubber molecule [6]. In the present study, unigenes encoding 4-hydroxy-3-methylbut-2-enyl diphosphate reductase (HDR, unigene25794), 1-deoxy-D-xylulose 5-phosphate synthase (DXR, unigene3946) that is components of MEP pathway, and encoding hydroxymethylglutaryl-CoA reductase (HMGR, unigene38121) and acetyl coenzyme A acetyltransferase (AACT, unigene14484) that is the key enzymes of MVA pathway were notably up-regulated in CATAS8-79 (Figure 6). Of which, unigene38121 for HMGR, unigene3946 for DXR, unigene25794 for HDR were analyzed by qRT-PCR (Figure 5). Additionally, unigenes encoding farnesyl diphosphate synthase (FDPS, unigene33714), geranyl-diphosphate synthase (GPPS, unigene21852 and unigene4306), *Hevea* rubber transferase 2 (HRT2, Unigene37088) which directly participated in the formation of the final high-molecular weight rubber molecule were also differentially expressed (Figure 6). FDPS and HRT2-related unigenes were up-regulated in CATAS8-79 whereas GPPS-related unigene was up-regulated in PR107, respectively (Figure 6). Jasmonates were pivotal to the secondary laticifer differentiation while ethylene was most effective in prolonging the duration of latex flow upon tapping in rubber tree [32,33]. In the present study, the up-regulated unigenes in JA signaling pathway in CATAS8-79 included unigene 34101 (*lipoxygenaseA, LOX*), 38638 (*lipoxygenaseA, LOX*), 24544 (*lipoxygenaseA, LOX*), 15932 (12-oxophytodienoate reductase3*, OPDR*), 40284 and 39621 (*jasmonate O-methyltransferase, JMT*), and 38882 (*Jasmonate ZIM-domain, JAZ8*). And the up-regulated unigenes in ethylene signaling pathway were unigene 23152 (*1-aminocyclopropane-1-carboxylate oxidase homolog 1, ACO*), 40400 (*1-aminocyclopropane-1-carboxylate oxidase, ACO*), 20082 and 17548 (*ethylene-overproduction protein 1*), 24000 and 20993 (*ethylene-insensitive protein 2*), 6206 and 17574 (*EIN3-binding F-box protein 1*) (Figure 6). Of which, unigene 38638 for LOX and unigene 23152 for ACO were analyzed by qRT-PCR (Figure 5). Whereas five of six unigenes (22954, 1657, 34623, 51430, 34785) encoding ethylene response factors were up-regulated in PR107. In addition, antioxidants antagonized reactive oxygen species -caused membrane lipid peroxidation and had a role in keeping the integrity of lutoid [16,17]. The unigene 18915 and 15723 encoding L-ascorbate peroxidase (APX) and superoxide dismutase (SOD) were up-regulated in CATAS8-79, respectively (Figure 5).

### Expression pattern of unigenes related to latex regeneration and duration of latex flow upon successive tapping

**Figure 7:**
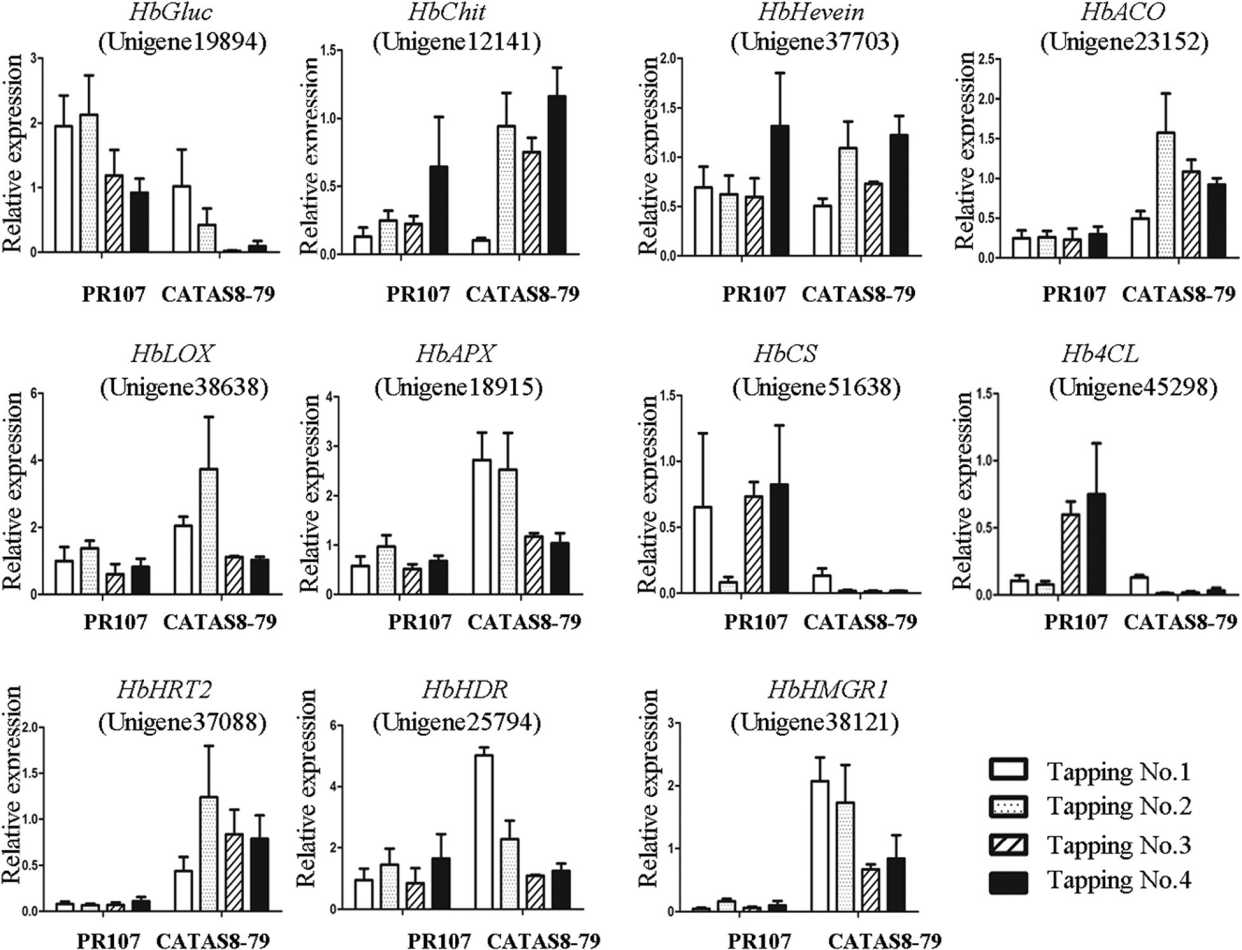
qRT-PCR analysis of the expression pattern of latex regeneration and expelling related genes upon successive tappings. Error bars for qRT-PCR showed the standard deviation of three replicates.

Eleven unigenes with differential expression between the two clones have been demonstrated to participate in latex regeneration or duration of latex flow. For this purpose, their expression patterns upon successive tapping were analyzed by qRT-PCR (Figure 7). Six of the eleven unigenes exhibited a big difference in their expression patterns between CATAS8-79 and PR107. Of which, unigene 49104 (*beta-1,3-glucanase, HbGluc*), 51638 (*cellulose synthase, HbCS*) and 45298 (*4-coumarate-CoA ligase, Hb4CL*) were up-regulated and kept significant high level in PR107 while similar expression pattern of unigene 23152 (*HbACO*), 38121 (*HbHMGR1*) and 37088 (*HbHRT2*) occurred in CATAS8-79 (Figure 7). The expression pattern of the other five unigenes was similar between the two clones (Figure 7).

## Discussion

The rubber tree clone PR107 is an original clone selected from Wickham germplasm in 1920s. The rubber tree clone CATAS8-79 was selected from hybrid offspring of CATAS88-13 and CATAS217. The CATAS88-13 is the product of RRIM600 and PilB84 cross while the CATAS217 is selected from the cross of RRIM513 and PR107. The RRIM600 are selected from the cross of Tjir1 and PB86 while RRIM513 is the hybrid offspring of PilB16 and PilA44. As there is a significant difference in duration of latex flow and latex regeneration between CATAS8-79 and PR107, analysis of the transcriptome dataset of PR107 against that of CATAS8-79 will be prospect to dissect key genes mediating the process. Some of the differentially expressed unigenes should be related to the difference in latex regeneration and duration of latex flow although the others may associate with the general difference in genetic background, considering that CATAS8-79 only contained one fourth descent of PR107.

It has long been believed that the efficiency of sucrose transportation and metabolism and rubber biosynthesis closely associates with the ability of latex regeneration between interval of successive tappings [7]. Amylase, a member of glycosyl hydrolases, is activated at weakly alkaline pH [34,35]. One of the effects of ethrel is to enhance carbohydrate metabolism by alkalizing cytosol of laticifer cells [36], which may be ascribed to the activation of amylase. Besides, ethrel treatment can greatly up-regulate the expression of SUT genes in rubber tree [37]. In the present study, several unigenes encoding beta-amylases and SUTs are found to be expressed at higher level in CATAS8-79 than that in PR107, suggesting a more efficiency of sucrose transportation and carbohydrate metabolism occurred in CATAS8-79 (Figure 6). IPP is biosynthesized though both MVA and MEP pathway and runs several enzymatic reactions to form the final high-molecular weight rubber molecule [7]. In present study, five unigenes encoding for HMGR and six unigenes encoding for HDR are detected with differently expression between two clones. Further sequence analysis shows four HDR-like members (unigene 4972, 25794, 8293, 37446) are perfectly blasted to one *HbHDR* with a Genbank number EU881977 whereas five HMGR-like unigenes are matched to three *HbHMGR* genes (unigene11873, unigene29798 for *HbHMGR4*, unigene28394, unigene38121 for *HbHMGR1*, unigene2975 for *HbHMGR5*) respectively. Previous work shows that the transcripts of *HbHMGR1* are most abundant in latex [38]. The present study shows that HMGR1-like unigenes are significantly up-regulated in CATAS8-79 while *HbHDR* displays a similar pattern between the two clones, suggesting HMGR1 in MVA pathway is critical for providing IPP.

Post-IPP processes include initiation and elongation of rubber macromolecules. FDPS family catalyzes the biosynthesis of farnesyl diphosphate (FDP). The FDP acts as the prime which is essential for initiating prenyl chain whereas HRT family is crucial for integrating IPP units into prenyl chain [39,40]. Two members of HRT family (HRT1 and HRT2) are reported in rubber tree [41]. *In vitro* analysis shows that only HRT2 has rubber transferase activity and may play a key role in extending prenyl chain [41]. In this study, unigenes related to HRT2 and FDPS are found to be expressed at higher level in CATAS8-79 than that in PR107, indicating that HRT2 and FDPS are crucial for enhancing rubber initiation and elongation. The higher expression level of HRT2 and FDPS like unigenes is in line with the higher rubber yield per tapping (Figure 1).

Jasmonate signaling plays a pivotal role in activating the secondary laticifer differentiation and activating the biosynthesis of secondary metabolites [42-45]. Although several members of COI1-JAZ-MCY module have been characterized in rubber tree and JA signaling is suggested to have an important role in regulating rubber biosynthesis in laticifer cells [46-48], the difference in the level of endogenous JAs in laticifer cells among *Hevea* germplasm remains largely unknown. The present study suggests that the level of endogenous JAs may be higher in the laticifer cells of CATAS 8-79 than that in the laticifer cells of PR107, considering that the expression of unigenes encoding enzymes such as LOX, OPDR, JMT is significantly up-regulated in CATAS8-79.

Ethrel, an ethylene releaser, is very effective in prolonging duration of latex flow [32]. One of the explanations for ethrel-induced prolongation of latex flow is its effect on maintaining the turgor pressure of laticifer and surrounding liber cells via its differential regulation on the aquaporins on the plasma membrane and tonoplast [49]. Considering that unigenes such as ACO, Ethylene overproduction protein and EIN3-binding F-box protein are up-regulated in CATAS8-79, the activity of ethylene biosynthesis and signal transduction in the laticifer cells of CATAS 8-79 may be higher than that of PR107. Besides, such factors as laticifer turgor, antioxidants, inclusions and debris of lutoid, and proteins in C-serum are suggested to influence the duration of latex flow [10-12]. After tapping, the bursting of lutoid particles leads to the release of hevein, chitinase, and glucanase. These protein inclusions are effective in rubber particle aggregation [5]. In the present study, it is the unigene encoding for glucanase other than the unigenes encoding for hevein and chitinase that is significantly up-regulated in PR107, suggesting that glucanase is more important in inducing rubber particle aggregation. The turgor pressure of laticifer in CATAS8-78 is significantly higher than that in PR107 [50]. In the present study, the unigenes enconding for enzymes (HbCS; Hb4CL) of cellulose and lignin biosynthesis are highly activated in RP107. This suggests that difference in turgor pressure may primarily associate with the difference in lignin content of laticifer cell wall between the two clones.

## Conclusions

**Figure 8:**
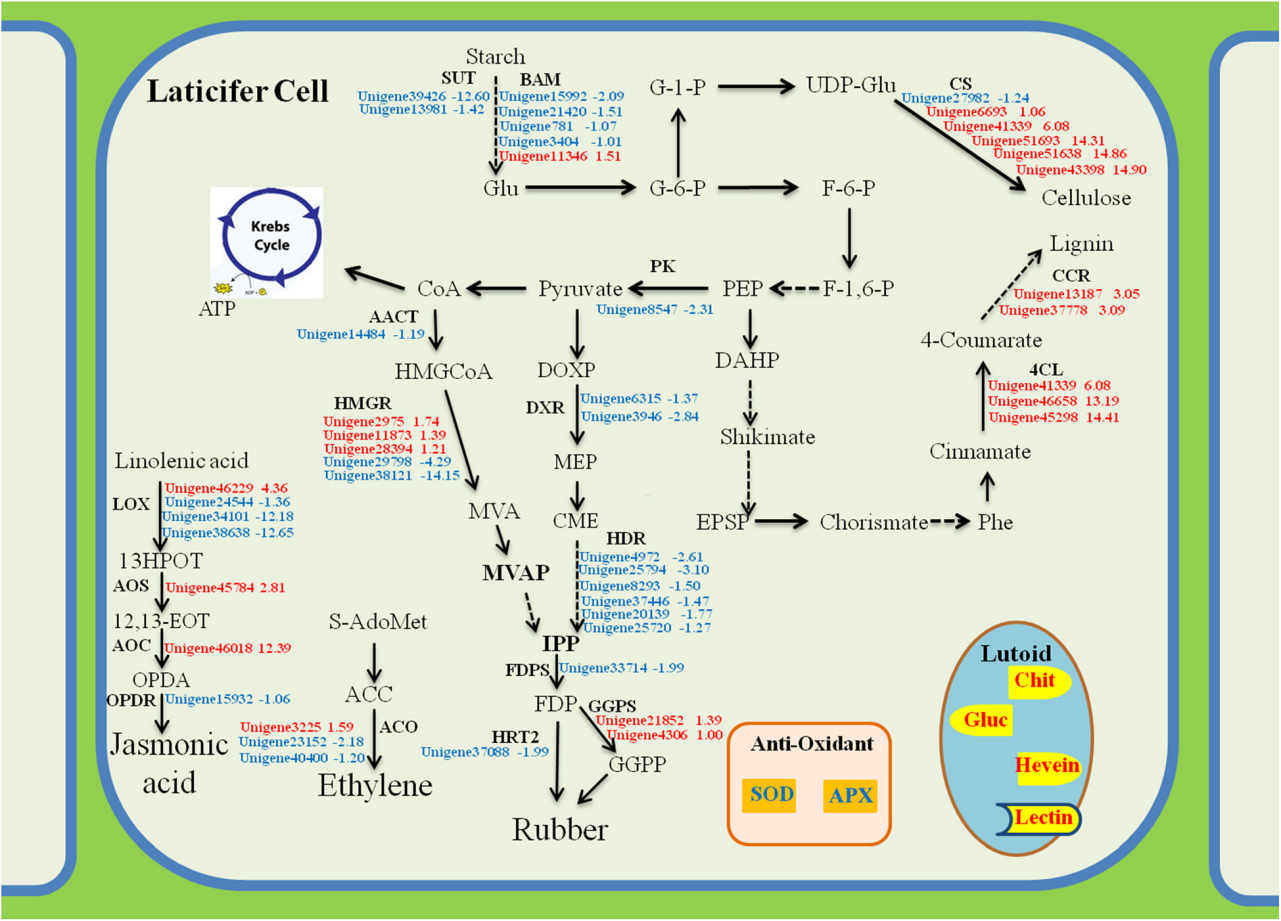
Schematic representation of differentially expressed unigenes related to latex regeneration and duration of latex flow in laticifer cell. Red colors showed unigenes up-regulated in PR107 while down-regulated in CATAS8-79. Blue colors showed unigenes up-regulated in CATAS8-79 while down-regulated in PR107. Numbers represented Log2 values in DGE data. The dashed arrows indicated multiple steps of enzymatic reactions.

Taken together, comparative transcriptome analysis reveals new cues at molecular level for the difference in duration of latex flow and latex regeneration between rubber tree clone CATAS8-79 and PR107 (Figure 8). Up-regulated expression of unigenes *HbLOX* and *HbOPDR* in the pathway of JA biosynthesis, *HbSUT, HbBAM* and *HbPK* mediating carbohydrate metabolism, *HbHMGR1* in MVA parthway and *HbHR*T*2* directly in rubber biosynthesis is important for enhanced latex regeneration. It may be essential for prolonging duration of latex flow that up-regulated expression of unigenes *HbACO* mediating ethylene biosynthesis, *HbAPX* and *HbSOD* for antagonizing reactive oxygen species, but down-regulated expression of unigenes *Hb4CL* and *HbCCR* in the pathway of lignin biosynthesis and *HbGluc* for rubber particle aggregation.

## Methods

### Plant materials

Seven-year-old virgin trees of rubber tree clone CATAS8-79 and PR107 were grown at the Experimental Station of the Rubber Research Institute of the Chinese Academy of Tropical Agricultural Sciences in Danzhou city, Hainan province, P.R. China. The virgin trees with same circumference were seleced in this study. For RNA-Seq, latex from five individual trees by the first tapping was pooled for each clone. The samples were immediately stored at -80°C until RNA extraction. For real time-PCR and determination of physiological parameters, latex was individually collected from another batch of five trees for each clone upon the first, second, third and forth tapping, respectively. All the selected virgin trees were tapped with a tapping system of S/2, d/2 (a half spiral pattern, every two days) at 6:00 am in August, 2013.

### Rubber content determination

To determine rubber content of latex, 100μl of acetic acid were dropped into 1g of fresh latex to obtain rubber coagula. The sample rubber coagula were washed in water for 2 h, then dried overnight at 55°C and weighted. The experiments were repeated three times.

### RNA isolation and sequencing

Total latex RNA was extracted as described [7] and RNA integrity was evaluated by NanoDrop (Thermo Scientific Inc., USA). The double strand cDNA was synthesized using SuperScript® Double-Stranded cDNA Synthesis Kit (Invitrogen Inc., USA), and purified and added single nucleotide A (adenine) to the end with QiaQuick PCR extraction kit. Finally, sequencing adaptors were ligated to the cDNA fragments. The required fragments were purified by 2% agarose gel electrophoresis and enriched by PCR amplification. The library products were sequenced via Illumina HiSeq™ 2000 by Beijing Genomics Institute (Shenzhen, China). The original image datasets was transferred into sequence datasets by base calling. Clean reads were obtained by removing adaptor sequence, low quality sequences, empty tags, low complexity, and tags with only one copy.

### Transcriptome de nove assembly, annotation and classification

Transcriptome *de novo* assembly was carried out using a *de* Bruijn graph and the SOAPdenovo as previously described [26]. Under a certain overlap length (k-mer = 29), SOAPdenovo combined overlapping reads into contigs. Adjacent contigs were constructed into scaffolds by read mate pairs. Within the scaffold, the connected contigs used ‘N’ to represent unknown sequences and insert size information. Finally, paired-end information was used to fill the gap of scaffolds to obtain the extended sequences with fewer Ns, which were defined as unigenes for further analysis.

All unigenes were used for BLAST searches (E-value < 1E-5) against databases as NCBI Nr (http://www.ncbi.nlm.nih.gov/), Swissprot (http://www.expasy.ch/sprot/), KEGG (http://www.genome.jp/kegg/) and COG (http://www.ncbi.nlm.nih.gov/cog/). The best aligning results were chosen for unigene annotation. The aligning results were selected with an order of Nr, Swiss-Prot, KEGG and COG.

To classify the unigenes, the Blast2GO program was used to get GO annotation based on molecular function, biological process and cellular component. All unigenes were also aligned to the COG database to predict possible functions and KEGG pathway database to perform pathway assignments.

### Digital gene expression analysis

A rigorous algorithm was developed to identify differentially expressed genes between two different DGE libraries (CATAS8-79 versus PR107). Raw clean tags in each library were normalized to Tags Per Million (TPM) to obtain normalized gene expression level. Differential digital gene expression was deemed with FDR value ≤0.001 and |log2 Ratio| ≥ 1 in sequence counts across libraries. “Up-regulated” means the level of gene transcripts were higher in PR107 whereas “down-regulated” means the level of gene transcripts were higher in CATAS8-79.

### Quantitative PCR analysis

Approximately 1 μg of RNA was used for reverse transcription based on the introduction of RevertAid™ First Strand cDNA Synthesis Kit (Thermo Scientific Inc., USA). qPCR was performed on the CFX96 System (Bio-Rad Laboratories Inc., USA) with SYBR PrimeScript RT-PCR Kit (TaKaRa Biotechnology, Japan). Nine housekeeping genes (*Hb18s, HbActin, HbELF1A, HbRH2B, HbRH8, HbYLS8, HbUBC2A, HbUBC2B, HbUBC4*) were selected to evaluate the stability by the software package NormFinder (version 0.953, http://www.mdl.dk/publicationsnormfinder.htm) [51]. *HbUBC2B* and *HbUBC4* were suitable for reference genes in the present study due to its stability in latex samples upon tapping (Additional file 3: Figure S2). Expression values were normalized for differences in cDNA input using parallel reactions employing primers designed against a reference gene *HbUBC2B* (HQ323247). All primer pairs used in this article were list in as an Additional file 4: Table S2.

### Statistical analyses

The statistical analyses were ANOVAs carried out on raw data. A *t* test (Student’s *t* test) was carried out for the significance analysis. *** indicates very significantly difference (p < 0.01).

### Availability of supporting data

The authors confirm that all data underlying the findings are fully available without restriction. Data are available at the following URL: http://www.ncbi.nlm.nih.gov/geo/query/acc.cgi?acc=GSE59981.

## Additional files

Additional file 1: Table S1. Statistics of DGE sequencing from CATAS8-79 and PR107 libraries. Additional file 2: Figure S1. Species distribution of unigenes with matches in Nr (A) and Swissport (B) databases. The species distribution is shown as a total homologous sequences in the NCBI Nr (A) and Swissport (B) databases with an E-value < 10 − 5. Additional file 3: Figure S2. Average expression stability values of the candidate reference genes evaluated by NormFinder. The least stable genes are on the left, and the two most stable genes are on the right. Additional file 4: Table S2. Primers used in this paper.

## Abbreviations

4CL: 4-coumarate-coa ligase
ACO: 1-aminocyclopropane-1-carboxylate oxidase
AOS: Allene oxide synthase
AOC: Allene oxide cycalse
BAM: Beta-amylase
CCR: Cinnamoyl-CoA reductase
Chit: Chitinase
COG: Clusters of Orthologous Group
CS: Cellulose synthase
DXR: 1-deoxy-D-xylulose 5-phosphate reductoisomerase
DGE: Digital gene expression
FDPS: Farnesyl diphosphate synthase
Gluc: Glucanase
GO: Gene Ontology
GPPS: Geranyl-diphosphate synthase
GS: Glutamate synthase
HDR: 4-hydroxy-3-methylbut-2-enyl diphosphate reductase
HMGR: Hydroxymethylglutaryl-CoA reductase
HRT: Hevea rubber transferase
IPP: Isopentenyl diphosphate
JA: Jasmonic acid
JMT: Jasmonate O-methyltransferase
KEGG: Kyoto Encyclopedia of Genes and Genomes
LOX: Lipoxygenase
MEP: 2-C-methyl-D-erythritol 4-phosphate
MVA: Mevalonate
OPDR: 12-oxophytodienoate reductase
PK: Pyruvate kinase
APX: L-ascorbate peroxidase
qRT-PCR: Quantitative reverse transcription-PCR
RPA: Rubber particle aggregation
SNP: Single nucleotide polymorphism
SOD: Superoxide dismutase
SUT: Sucrose transporter
