# Supplementary material for: Comparative transcriptome analysis of latex from rubber tree clone CATAS8-79 and PR107 reveals new cues for the regulation of latex regeneration and duration of latex flow
Source: BMC Plant Biol. 2015 Apr 18;15:104. doi: 10.1186/s12870-015-0488-3 (PMC4410575; doi:10.1186/s12870-015-0488-3)
Supplement: Additional file 1: Table S1. — Statistics of DGE sequencing from CATAS8-79 and PR107 libraries. [file 12870_2015_488_MOESM1_ESM.doc]

**Additional file 1**

**Table S1. Statistics of DGE sequencing for CATAS8-79 and PR107 libraries**

| Sample | Number  /percent | 100-500nt | 500-1000nt | 1000-1500nt | 1500-2000nt | >2000nt | N50 | Mean (bp) | No. | Length(bp) |
| --- | --- | --- | --- | --- | --- | --- | --- | --- | --- | --- |
| **CATAS8-79 Contig** | number | 296,736 | 10,351 | 1,649 | 531 | 261 | 133 | 142 | 305,004 | 43,311,050 |
|  | percent | 95.87% | 3.34% | 0.53% | 0.17% | 0.08% |  |  |  |  |
| **PR107 Contig** | number | 308,262 | 9,932 | 1,396 | 360 | 129 | 124 | 137 | 315,643 | 43,387,443 |
|  | percent | 96.31% | 3.10% | 0.44% | 0.11% | 0.04% |  |  |  |  |
| **CATAS8-79 Unigene** | number | 41,457 | 8,474 | 2,224 | 824 | 592 | 509 | 421 | 53,571 | 22,572,807 |
|  | percent | 77.39% | 15.82% | 4.15% | 1.54% | 1.11% |  |  |  |  |
| **PR107**  **Unigene** | number | 46,999 | 8,243 | 1,744 | 539 | 281 | 427 | 375 | 57,806 | 21,689,990 |
|  | percent | 81.30% | 14.26% | 3.02% | 0.93% | 0.49% |  |  |  |  |
| **All**  **Unigene** | number | 35,195 | 11,019 | 3,323 | 1,277 | 1,015 | 640 | 526 | 51,829 | 27,237,155 |
|  | percent | 67.91% | 21.26% | 6.41% | 2.46% | 1.96% |  |  |  |  |
|  |  |  |  |  |  |  |  |  |  |  |
