# Supplementary figures and images for: Comparative transcriptome analysis of latex from rubber tree clone CATAS8-79 and PR107 reveals new cues for the regulation of latex regeneration and duration of latex flow
Source: BMC Plant Biol. 2015 Apr 18;15:104. doi: 10.1186/s12870-015-0488-3 (PMC4410575; doi:10.1186/s12870-015-0488-3)

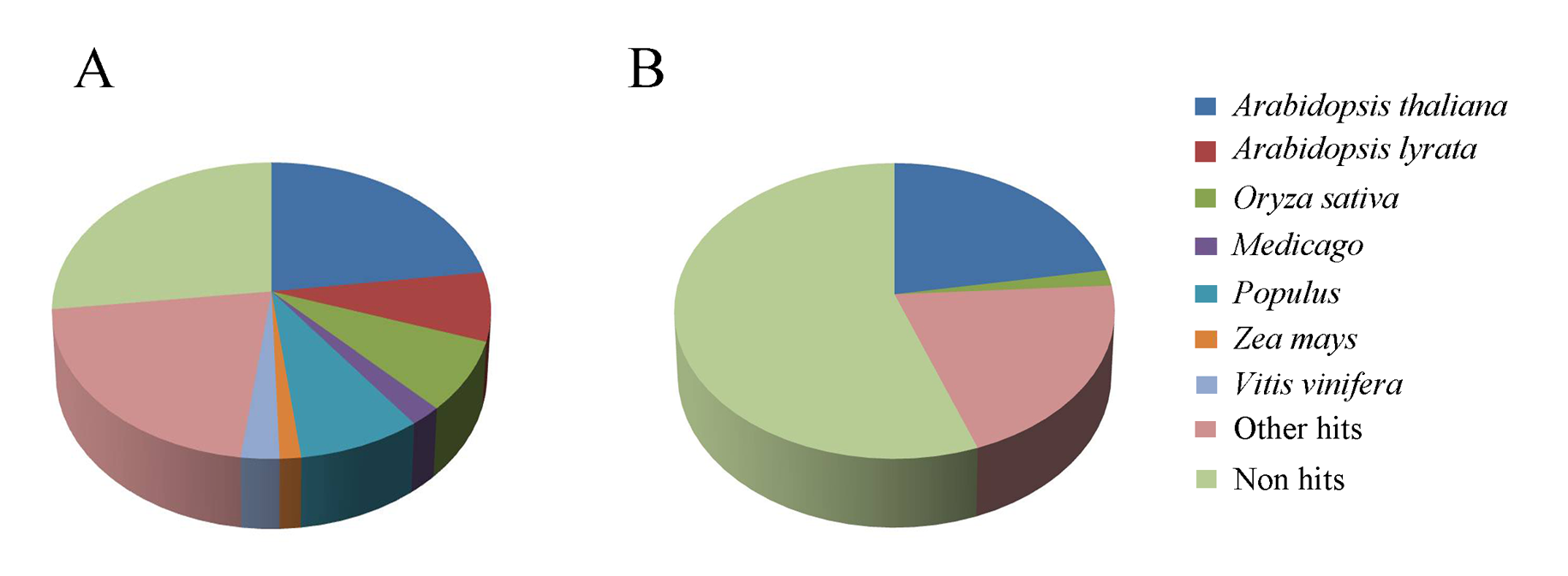

Supplement: Additional file 2: Figure S1. — Species distribution of unigenes with matches in Nr (A) and Swissport (B) databases. The species distribution is shown as a total homologous sequences in the NCBI Nr (A) and Swissport (B) databases with an E-value < 10 − 5. [file 12870_2015_488_MOESM2_ESM.tiff]

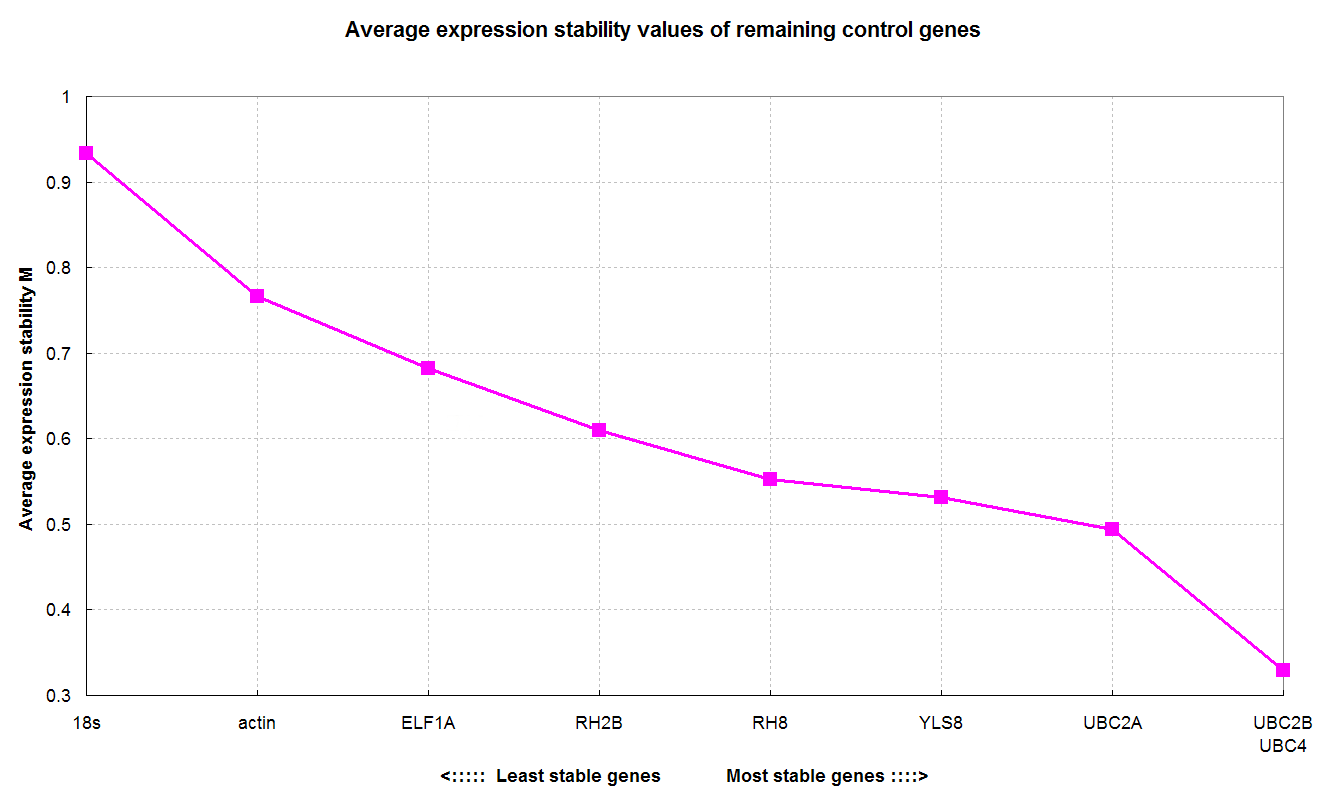

Supplement: Additional file 3: Figure S2. — Average expression stability values of the candidate reference genes evaluated by NormFinder. The least stable genes are on the left, and the two most stable genes are on the right. [file 12870_2015_488_MOESM3_ESM.tiff]
