# Supplementary material for: Comparative transcriptome analysis of latex from rubber tree clone CATAS8-79 and PR107 reveals new cues for the regulation of latex regeneration and duration of latex flow
Source: BMC Plant Biol. 2015 Apr 18;15:104. doi: 10.1186/s12870-015-0488-3 (PMC4410575; doi:10.1186/s12870-015-0488-3)
Supplement: Additional file 4: Table S2. — Primers used in this paper. [file 12870_2015_488_MOESM4_ESM.doc]

**Additional file 4**

**Table S2.** Primers used in this paper.

| **Unigene ID** | **Primer sequence** | **Annotation** |
| --- | --- | --- |
| **Unigene51625** | Forward: CATCTTCGTTTGGTGGTA  Reverse: CAACCCAACGCTCAAGTA | Protein BRASSINOSTEROID INSENSITIVE 1 |
| **Unigene51638** | Forward: GGCCACTTCTAAGGTAT  Reverse: TGCTAGTTGCAGCTATG | Cellulose synthase-like protein |
| **Unigene40609** | Forward: TTCCAGGGTCTCAATCAC  Reverse: AACCCATCCATAGCATCA | Auxin-responsive protein |
| **Unigene39426** | Forward: GGAGGGCTTACCACATT  Reverse: CTTCTCGGCCAACTTTT | Sucrose transporter |
| **Unigene38121** | Forward: GCCAGCGGCTAAGAAGAA  Reverse: ACCTCCGGGAATCCCATC | Hydroxymethylglutaryl-CoA reductase 1 |
| **Unigene25794** | Forward: CTTCGCGTCAAGTTATGT  Reverse: AACTCTGCCGCGTCTCC | 4-hydroxy-3-methylbut-2-enyl diphosphate reductase |
| **Unigene40284** | Forward: TCGGTCAAACACCTTACT  Reverse: CTCACCTTCCTCGCACAG | Jasmonate O-methyltransferase |
| **Unigene13187** | Forward: TCCTTCTGCGGGTCCTT  Reverse: TCCACCTCCTCCCTTCC | Cinnamoyl-CoA reductase |
| **Unigene38638** | Forward: TCTCCATGCAGCAATCA  Reverse: GGGAAATGCCAACAACA | Lipoxygenase |
| **Unigene12141** | Forward: GCACCACAGTAATCAGG  Reverse: TTGGCTTTCACAGTTTC | Chitinase |
| **Unigene37703** | Forward: GCTAGCTCAGATATGGTTGACTTCC  Reverse: TGATGATTCTTCACCCGTCAGCGTA | Hev b 7 |
| **Unigene34513** | Forward: AAGCCATAGTCATACCCA  Reverse: GACCAAATAGCGTCTGCA | Probable serine/threonine-protein kinase |
| **Unigene45298** | Forward: CCAGGCAACTAAGCAGA  Reverse: CAGGGTGAGAAACAAGC | 4-coumarate--CoA ligase |
| **Unigene49104** | Forward: AATCTGAAGAAACGGCTAT  Reverse: GTCCCGTGAATCCAATA | Beta-1,3-glucanase |
| **Unigene19894** | Forward: GGCTATCCTGGTCCTCA  Reverse: CCACCTGATACTCCCACT | Glutamine synthetase |
| **Unigene21420** | Forward: ACAGTGTAGAAATGCGAGGTT  Reverse: GCAGGATTCGGGAGAAA | Beta-amylase 2 |
| **Unigene39426** | Forward: GGAGGGCTTACCACATT  Reverse: CTTCTCGGCCAACTTTT | Sugar transporter |
| **Unigene51560** | Forward: CACGAGTCTTTGCCTGAG  Reverse: GGTCTTGAACTTCGCTTT | Disease resistance response protein |
| **Unigene23152** | Forward: ATCATCGAGCATAAACTG  Reverse: AAGCGTAGATCATAGAGTGT | 1-aminocyclopropane-1-carboxylate oxidase |
| **Unigene33714** | Forward: AGCCTATTTTCTTGTCC  Reverse: ATAGTATGCCTTCCCTC | Farnesyl pyrophosphate synthase |
| **Unigene3946** | Forward: CATTGTTGTTCTTCCCTAG  Reverse: TCAGGCATACCCACATA | 1-deoxy-D-xylulose 5-phosphate synthase |
| **Unigene37088** | Forward:TTTCTGTTCCTAGCTCATCCTGCCT  Reverse:TGCATGTCGGAATTTGCTACAATAC | Hevea rubber transferase 2 |
| **Unigene18915** | Forward: ATTGCTGATCCCGTCTT  Reverse: GTCGGCACCATCCTCTA | L-ascorbate peroxidase 2 |
| **Hb18S** | Forward: GCTCGAAGACGATCAGATACC  Reverse: TTCAGCCTTGCGACCATAC |  |
| **HbActin** | Forward: GATTCCGTTGCCCAGAAGTC  Reverse: CACCACTCAGCACAATGTTACC |  |
| **HbELF1A** | Forward: GCGTGACTATCAGGACGACAA  Reverse: CAAGACCTCCAGCAATACCCT |  |
| **HbRH2B** | Forward: CGACCAAGTTTTCATTTCGGGTG  Reverse: AGTCTCTTCTTTGCTGGGGTTG |  |
| **HbRH8** | Forward: TCACAGGGTTGGTAGATCAG  Reverse: CCAAGCTCTTGCTCAATCC |  |
| **HbYLS8** | Forward: CCTCGTCGTCATCCGATTC  Reverse: CAGGCACCTCAGTGATGTC |  |
| **HbUBC2A** | Forward: CATTTATGCGGATGGAAGCA  Reverse: CAGGGGAGTTTGGATTTGGA |  |
| **HbUBC2B** | Forward: CGACCAAGTTTTCATTTCGGGTG  Reverse: AGTCTCTTCTTTGCTGGGGTTG |  |
| **HbUBC4** | Forward: TCCTTATGAGGGCGGAGTC  Reverse: CAAGAACCGCACTTGAGGAG |  |
